# ICU admission body composition: skeletal muscle, bone, and fat effects on mortality and disability at hospital discharge—a prospective, cohort study

**DOI:** 10.1186/s13054-020-03276-9

**Published:** 2020-09-21

**Authors:** Ariel Jaitovich, Camille L. Dumas, Ria Itty, Hau C. Chieng, Malik M. H. S. Khan, Ali Naqvi, John Fantauzzi, Jesse B. Hall, Paul J. Feustel, Marc A. Judson

**Affiliations:** 1 Division of Pulmonary and Critical Care Medicine, Albany Medical College, Albany, NY USA; 2 Department of Molecular and Cellular Physiology, Albany Medical College, 47 New Scotland Av, Albany, NY USA; 3 Department of Radiology, Albany Medical College, Albany, NY USA; 4 Present Address: Division of Pulmonary and Critical Care Medicine Spectrum Health-Michigan State University College of Human Medicine, Grand Rapids, MI USA; 5 Section of Pulmonary and Critical Care, Department of Medicine, University of Chicago, Chicago, IL USA; 6 Department of Neuroscience and Experimental Therapeutics, Albany Medical College, Albany, NY USA

**Keywords:** Skeletal muscle, Adipose tissue, Bone density, Critical illness, Mortality

## Abstract

**Background:**

Reduced body weight at the time of intensive care unit (ICU) admission is associated with worse survival, and a paradoxical benefit of obesity has been suggested in critical illness. However, no research has addressed the survival effects of disaggregated body constituents of dry weight such as skeletal muscle, fat, and bone density.

**Methods:**

Single-center, prospective observational cohort study of medical ICU (MICU) patients from an academic institution in the USA. Five hundred and seven patients requiring CT scanning of chest or abdomen within the first 24 h of ICU admission were evaluated with erector spinae muscle (ESM) and subcutaneous adipose tissue (SAT) areas and with bone density determinations at the time of ICU admission, which were correlated with clinical outcomes accounting for potential confounders.

**Results:**

Larger admission ESM area was associated with decreased odds of 6-month mortality (OR per cm^2^, 0.96; 95% CI, 0.94–0.97; *p* < 0.001) and disability at discharge (OR per cm^2^, 0.98; 95% CI, 0.96–0.99; *p* = 0.012). Higher bone density was similarly associated with lower odds of mortality (OR per 100 HU, 0.69; 95% CI, 0.49–0.96; *p* = 0.027) and disability at discharge (OR per 100 HU, 0.52; 95% CI, 0.37–0.74; *p* < 0.001). SAT area was not significantly associated with these outcomes’ measures. Multivariable modeling indicated that ESM area remained significantly associated with 6-month mortality and survival after adjusting for other covariates including preadmission comorbidities, albumin, functional independence before admission, severity scores, age, and exercise capacity.

**Conclusion:**

In our cohort, ICU admission skeletal muscle mass measured with ESM area and bone density were associated with survival and disability at discharge, although muscle area was the only component that remained significantly associated with survival after multivariable adjustments. SAT had no association with the analyzed outcome measures.

## Background

Skeletal muscle dysfunction, encompassing wasting and weakness [1, 2], is associated with poor intensive care unit (ICU) outcomes including worse survival [3], need for mechanical ventilation, and higher readmission rate [4–6]. In many ICU survivors, muscle dysfunction persists for years [6, 7], and we have recently reported that reduced pectoralis muscle mass at the time of ICU admission is independently associated with higher mortality during and after ICU care [8]. Even though functional capacity is highly influenced by muscle work, our data showed that admission muscle mass was not significantly associated with disability at hospital discharge [8]. Moreover, while muscle mass substantially contributes to normal body weight [9], the overweight state is largely driven by fat mass [10]. Currently, there is controversy as to whether obese-range weight paradoxically portends a better ICU prognosis [11–13] or not [14–16]. Our previous data indicated that subcutaneous fat mass at the 7th vertebral level measured at ICU admission was not significantly associated with ICU outcomes [8].

While previous research indicates that muscle mass and bone density decrease as a result of ICU admission [1, 17], there is no information regarding the association of critically ill patients’ baseline bone density with survival and other patient-centered outcomes. A disaggregated evaluation of body constituents could improve our understanding of their potential pathophysiological contribution to critical illness outcomes.

In the present study, we extended our prior study [8] by analyzing the association of erector spinae muscle, fat mass, and bone density determined within 24 h of ICU admission with survival and disability at hospital discharge. We chose to measure the antigravity erector spinae muscle (ESM) area given that it is indispensable for upright posture and locomotion, and thus could be associated with both outcome measures, and specifically clarify the association of muscle mass with disability at hospital discharge. Our central hypothesis was that greater admission muscle mass would be the main driver of better ICU outcomes compared with a relatively less impactful effect of bone density and fat mass. To test this hypothesis, muscle mass was determined by the ESM cross-sectional area [18], fat was determined by measuring the subcutaneous adipose tissue (SAT) area at the thoracic 7–8th level areas previously established [19], and bone density was analyzed based on 12th vertebral body attenuation [20]. We also collected follow-up data on the patients’ clinical outcomes over time. For the analyses of fat mass, the previously published cohort was expanded with 104 patients.

## Materials and methods

This was a prospective, single-center, observational study of adult subjects admitted to the medical intensive care unit (MICU) of Albany Medical Center. Ethical approval was obtained from Albany Medical College Committee on Research Involving Human Subjects (IRB# 4281). Enrollment took place between November 2015 and June 2019, and the 6-month survival determination was completed in January 2020. Patients were considered for enrollment if they were older than 18 years, admitted to the MICU, had undergone a chest or abdomen CT scan at admission or were expected to undergo one within the first 24 h of MICU stay, and were anticipated to require ICU care for longer than 24 h. Exclusion criteria were primary neuromuscular pathology, acute illness leading to imminent death or chronic illness with a life expectancy of shorter than 6 months. Enrollment was attempted on all eligible patients, and written consent was obtained from the patient or a legally authorized representative. Prehospital comorbidities were determined by clinical history and with hospital documentation. Functional independence before admission was defined as the subject’s ability to live at home without help. Patients who were living at home with a caregiver, at a nursing home, or at an assisted living facility or who were transferred from a different institution were considered not functionally independent before hospitalization.

Our previous research [8] indicated that 100 events would need to occur to reach a significant association of muscle cross-sectional area with mortality at 6 months [8], which led to a targeted cohort size of 500 patients given a mortality rate of 30%, a 90% follow-up efficiency, and an estimate that 20% of CT scans would not be technically evaluable. Moreover, as in our previous report, we appreciated an underrepresentation of female sex and given that fat area was found significantly larger in these individuals [8]; here, we calculated that 220 female subjects would power the cohort similarly to the male’s arm in the previous study regarding the fat area analysis; that number would be achieved, assuming the same proportion of females (44%), by reaching a target cohort size of 500 participants. Enrolled patients were monitored daily for survival, length of ICU stay, and disposition at discharge. Survival at 6 months was determined either by hospital documentation or phone communication. If 6-month survival could not be determined by these methods, we searched for notifications of patients’ death in obituary lists published by local newspapers. To compare the enrolled versus non-enrolled patient populations during the study period, deidentified information including demographic characteristics and the reason for hospitalization of the entire MICU census was provided by the Albany Medical Center Department of Analytics following an IRB waiver. Out of the 507 patients enrolled in this cohort, 403 had been reported in a previous publication [8]; however, the erector spinal muscle and bone CT measurements obtained in this trial had not been previously analyzed or reported.

**Fig. 1:**
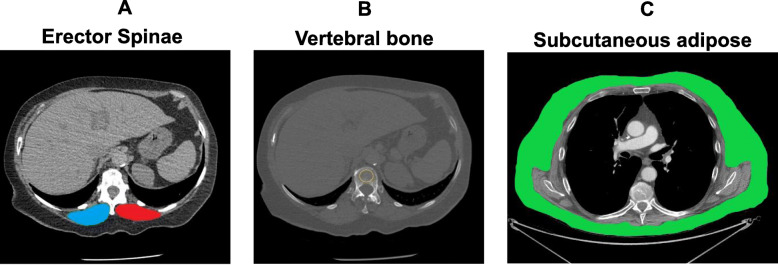
Sample computed tomography (CT) scans used to determine muscle area in our cohort. **a** Erector spinae muscle cross-sectional area (ESMCSA) measured at the T12 level: blue indicate right muscle and red, left muscle. **b** Bone density measured in the region of interest (ROI) indicated with yellow circle, at the T12 level. **c** Subcutaneous adipose tissue (SAT) measured at the T7-8 level, highlighted in green

Erector spinae muscle (ESM) area measurement was performed as previously established [18] using a single axial slice of the CT scan with an in-house software. In short, a single chest radiologist blinded to the patients’ identity and clinical characteristics visually identified the left and right ESMs which were manually shaded, and the ESM cross-sectional area (CSA) was presented as the sum of the right and left muscles expressed in square centimeters (Fig. 1a). Bone density was measured at the T12 level by determining the Hounsfield attenuation units (HU) value as previously established [20] (Fig. 1b), and subcutaneous adipose tissue was measured at the T7-8 vertebral level as previously described [19] (Fig. 1c). These tomographic levels were selected as they simultaneously capture the three body constituents of interest, which can be analyzed with previously described methods and accessed with either chest or abdomen CTs. A more detailed explanation of methods can be found in the supplementary material.

### Statistical analysis

The primary outcome variable was 6-month survival and the secondary outcome variable was disability at hospital discharge. We chose covariables based on previous literature and clinical relevance, and then assessed how they associated with categorical outcome measures using logistic regression models. Outcome-associated variables evaluated on a univariate basis and used in the multivariable model were (1) sex, (2) age, (3) SOFA score, (4) APACHE II score, (5) bone density, (6) fat area, (7) muscle area, (8) mMRC, (9) serum albumin, (10) functional independence before hospitalization, (11) pre-existing cancer, (12) pre-existing pulmonary disease, (13) pre-existing congestive heart failure, (14) pre-existing end-stage renal disease in dialysis, and (15) chronic steroid use. For multivariable models, we used a backward elimination approach with alpha to remove set at 0.10. Variance inflation factors (VIFs) were used to assess multicollinearity. Survival analysis was conducted on quartiles of ESM area using the log-rank test. In addition, the association of ESM with survival time was analyzed by using a Cox proportional hazard model using the same univariate and multivariable approach. Univariate and multivariable logistic regression and survival analysis were performed using Minitab Statistical Software (State College, PA) and JMP (SAS, Cary, NC), with significance accepted at a two-tailed alpha of 0.05.

Disability at discharge was analyzed using binary logistic regression with discharge location as the two possible outcomes: (1) discharge to a facility or home with assisted living required (discharged not independent) or (2) discharge to home without assisted living required (discharged independent). Comparison of ESM areas between these two groups was made by Mann-Whitney test. Because some patients’ CT scans were not analyzable for specific body constituents, univariate analyses were conducted for cases with complete data (*N* = 422, 463, and 423 for muscle, fat, and bone measurements, respectively). Multivariable analyses were conducted only using cases with complete data for all variables (420 cases).

## Results

**Fig. 2:**
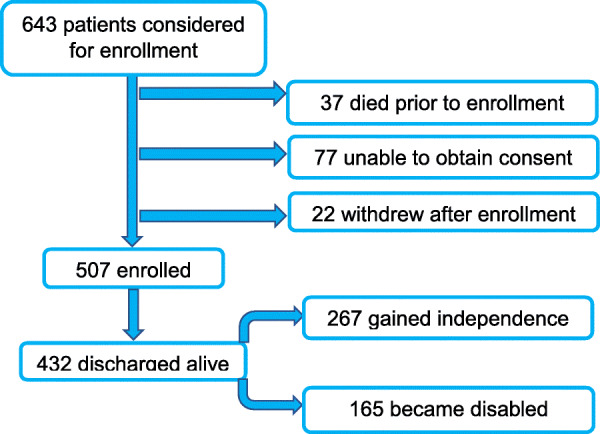
Enrollment flowchart

A total of 643 MICU patients were considered eligible for this study, of which 507 (79%) were included and 136 (21%) were excluded (Fig. 2). Sixty-five patients were later excluded for muscle analysis, 24 for fat analysis, and 63 for bone analysis due to technical limitations in determining these measures on CT leaving 422 cases for muscle, 463 cases for fat, and 423 for bone measurements. Four hundred and twenty cases had all three measures completed. The demographic data are shown in Table 1. The median ESM area was 30 cm^2^ with an interquartile range of 22.4–38.4. The median SAT was 16.7 cm^2^ with an interquartile range of 11–26; and the median bone density was 156 HU with an interquartile range of 118–195. The average age of the cohort was 62 years and there was a slight male predominance (54%). The unadjusted raw ESM area was smaller in females than males and weakly inversely correlated with age (Table E1). Similarly, a statistically significant negative correlation between bone density and age was found in this cohort (Table E1), which was consistent in magnitude with previously described data [20–22]. Out of the 507 patients enrolled and analyzed, 432 (85%) were discharged alive. Among these survivors, 267 (62%) were functionally independent and 165 (38%) were not functionally independent at discharge. At 6 months, 317 patients (62%) were alive, 168 (33%) had died, and 22 (5%) could not be reached to determine status. Although data of these 22 missing patients was obtained to count the number of cases with computed tomography, it was not included in the 6-months survival analyses (Table E2).

**Table 1:** Baseline characteristics of the cohort

**Demographics**		**IQR**
Male gender	54%	NA
Muscle area*	30	22.4–38.4
Fat area	16.7	11–26
Bone density	156	118–195
Age	62	51–72
**Severity Scores**		
APACHE II	16	11–22
SOFA	5	3–8
**Preadmission comorbidities**	**Total**	**%**
Pulmonary disease	170	35
Diabetes	121	25
Steroids use	112	23
Cancer	106	22
Congestive heart failure	90	19
End-stage renal disease	37	7.6
Preadmission mMRC**	3	1–4
**Primary reason of ICU admission**	**Total**	**%**
Respiratory failure	194	40
Non-respiratory sepsis	92	19
Pulmonary embolism	70	14
Hemorrhagic shock	40	8
Altered mental status	23	4.7
Metabolic cause including DKA***	16	3
Cardiovascular decompensation	16	3
Trauma	11	2
Other	21	4.5
**Residence before ICU admission**	**Total**	**%**
Living at home independently	261	54
Living with caregiver	64	13
Transferred from another hospital	60	12
Living at assisted living facility	37	7
Living at hospice	33	6
Unknown	28	5

*Median value of ESM; females’ areas were multiplied by 1.67 in this analysis

**mMRC, modified medical research council dyspnea score interquartile range (IQR)

***DKA, diabetic ketoacidosis

Total number of patients and percentages are calculated based on 483 cases, which is the number of CT scans that could be used for adipose tissue analysis. Notice that these figures were slightly different in muscle and bone measurements due to technical limitations in these determinations (see main text for details). “Preadmission comorbidities” is a category that admits more than one item per patient; reason for admission is a category that only admits one item per patient. *APACHE* Acute Physiology and Chronic Health Evaluation II score, *SOFA* Sequential Organ Failure Assessment score

### Association of admission ESM area with outcomes

**Fig. 3:**
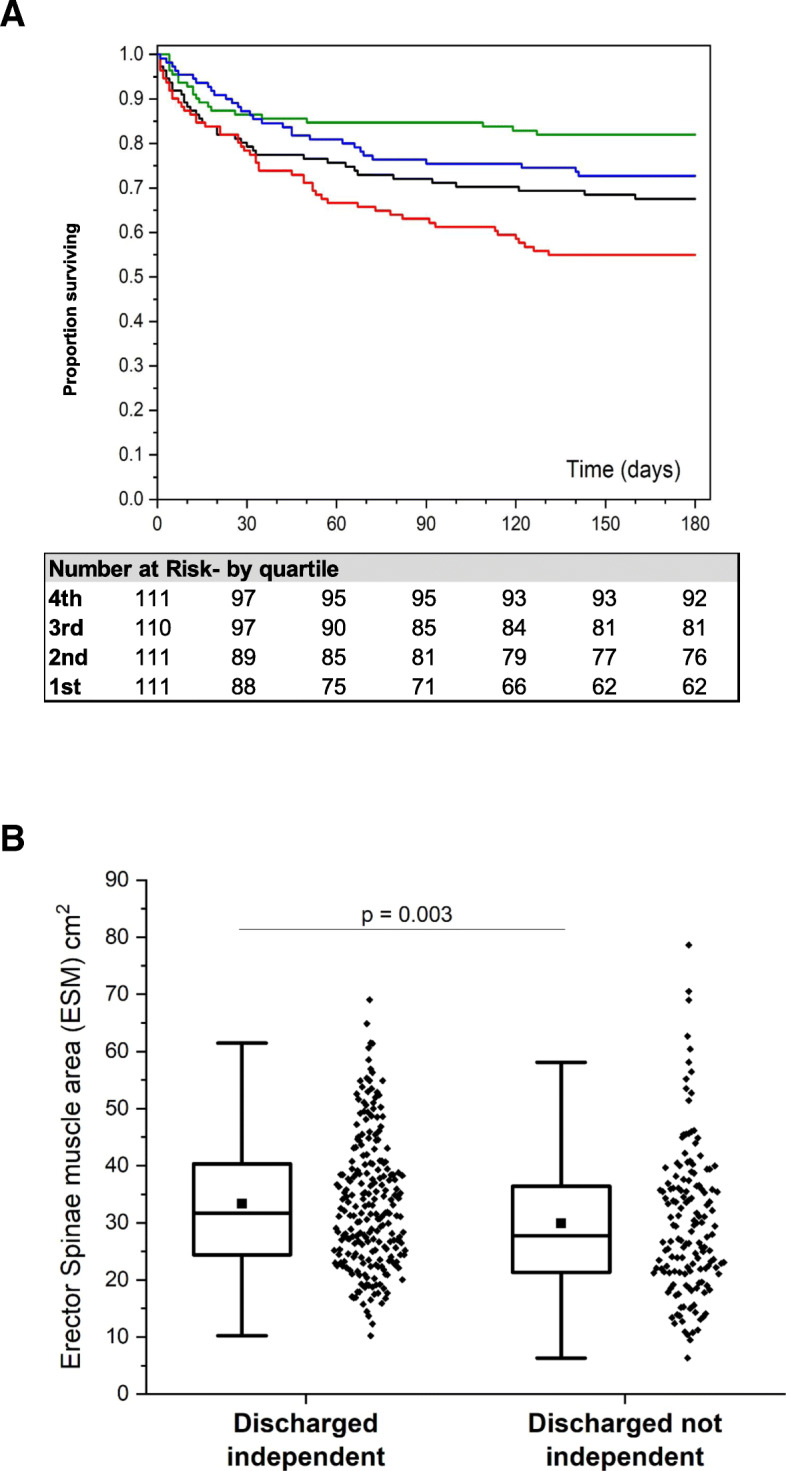
**a** Kaplan-Meier survival at 6 months based on ESCSA. Comparison of survival rate between patients with erector spinae muscle (ESM) cross-sectional area below divided in 4 quartiles. 1st quartile, red; 2nd quartile, black; 3rd quartile, blue; and 4th quartile, green (*p* < 0.001; log-rank test). At 6 months, 317 patients were alive, 168 had died, and 22 could not be reached to determine status. Female ESM was multiplied by 1.67 in this analysis (see supplement for details). **b** Distribution of erector spinae muscle CSA (in cm^2^) stratified by disposition at discharge. Comparison between groups was done with non-parametric Mann-Whitney test (*p* = 0.003). The groups are different with discharged independent (*n* = 231) greater than not independent (*n* = 150). See definition of discharged independent and not independent in the “Materials and methods” section. Female ESM area was multiplied by 1.67 in this analysis. For box plots, center line is median, upper and lower lines are 75th and 25th percentile, and whiskers are the non-outlier range (< 1.5 IQR from box)

Larger ESM was significantly associated with decreased odds of mortality at 6 months (OR 0.96 per cm^2^ increase in ESM; 95% CI 0.94–0.97; *p* < 0.001; Table 2). Figure 3a shows Kaplan-Meier curves for patients with muscle areas values divided in 4 quartiles (*p* < 0.001; log-rank test). In a multivariable analysis, the association of ESM with mortality persisted even after adjusting for other variables including severity scores, albumin, and mMRC (Table 2). ESM was associated with survival using a Cox proportional hazard model on a univariate basis (RR 0.96 per cm^2^ of muscle area, CI 0.95–0.98, *p* < 0.001) and in a Cox multivariable model (RR 0.98 per cm^2^ of muscle, 95%CI 0.97–0.999 *p* = 0.04; Table E3).

**Table 2:** Univariate and multivariate associations of analyzed covariables with 6 months survival (primary outcome) and disability at hospital discharge (secondary outcome)

**Variable (per unit change unless otherwise indicated)**	**Odds ratio**	**95% CI**	***p***
Age (per decade)	1.29	(1.13, 1.46)	**< 0.001**
Albumin	0.33	(0.24, 0.45)	**< 0.001**
SOFA	1.19	(1.13, 1.26)	**< 0.001**
APACHE II	1.10	(1.07, 1.13)	**< 0.001**
Bone density (per 100 HU)	0.69	(0.49, 0.96)	**0.03**
ESMCSA (muscle area, per cm^2^)	0.96	(0.94, 0.97)	**< 0.001**
Fat (per 10 cm^2^)	0.99	(0.97,1.01)	0.19
Sex M/F	1.39	(0.95, 2.03)	0.09
mMRC	1.36	(1.20, 1.55)	**0.011**
Not independent at admission	1.52	(1.03, 2.24)	**0.04**
Cancer	4.00	(2.57, 6.24)	**< 0.001**
Congestive heart failure	1.36	(0.85, 2.16)	0.2
Chronic pulmonary disease	1.12	(0.76, 1.65)	0.56
End-stage renal disease	2.11	(1.09, 4.08)	**0.03**
Chronic steroids	1.09	(0.71, 1.70)	0.69
**Multivariable risk factors for 6-month mortality (primary outcome)**			
**Variables included in the model**	**Odds Ratio**	**95% CI**	***p***
APACHE II	1.06	(1.01, 1.11)	**0.01**
SOFA	1.10	(1.01, 1.20)	**0.04**
Albumin	0.49	(0.33, 0.72)	**< 0.001**
ESMCSA (muscle area, per cm^2^)	0.98	(0.96, 0.99)	**0.03**
mMRC	1.20	(1.02, 1.42)	**0.03**
Cancer	3.25	(1.86, 5.69)	**< 0.001**
**Univariate associations for disability at discharge (secondary outcome)**			
**Variable (per unit change unless otherwise indicated)**	**Odds Ratio**	**95% CI**	***p***
Age (per decade)	1.45	(1.26, 1.66)	**< 0.001**
Albumin	0.51	(0.38, 0.69)	**< 0.001**
SOFA	1.11	(1.04, 1.17)	**0.001**
APACHE II	1.06	(1.03, 1.09)	**< 0.001**
Bone density (per 100 HU)	0.52	(0.37, 0.74)	**0.01**
ESMCSA (muscle area, per cm^2^)	0.98	(0.96, 0.99)	**0.012**
Fat (per 10 cm^2^)	1.01	(0.994, 1.03)	0.13
Sex M/F	0.76	(0.52, 1.13)	0.18
mMRC	1.40	(1.23, 1.60)	**< 0.001**
Not independent at admission	3.56	(2.35, 5.39)	**< 0.001**
Cancer	1.27	(0.78, 2.06)	0.34
Congestive heart failure	1.48	(0.90, 2.44)	0.13
Chronic pulmonary disease	1.25	(0.84, 1.87)	0.28
End-stage renal disease	1.18	(0.56, 2.47)	0.66
Chronic steroids	1.03	(0.65, 1.63)	0.91
**Multivariable risk factors for disability at discharge (secondary outcome)**			
**Variable**	**Odds Ratio**	**95% CI**	***p***
Age (per decade)	1.29	(1.09, 1.52)	**< 0.001**
APACHE II	1.04	(1.01, 1.08)	**0.02**
mMRC	1.18	(1.004, 1.40)	**0.045**
Albumin	0.56	(0.38, 0.83)	**< 0.001**
Not independent at admission	2.79	(1.71, 4.54)	**< 0.001**

Fat magnitude expresses subcutaneous adipose tissue (SAT) area; *ESMCSA* erector spinae muscle cross-sectional area, *mMRC* modified medical research council dyspnea score

Also, among survivors at hospital discharge, greater ESM was significantly associated with decreased odds of disability (OR 0.98 per cm^2^ ESM; 95% CI 0.96–0.99; *p* = 0.012; Table 2). Figure 3b compares muscle areas in those discharged functionally independent versus not functionally independent. Multivariable modeling indicated that the statistically significant association of muscle mass with disability at discharge was lost after adjusting for other significant covariables including severity score (Table 2).

### Association of admission bone density with outcomes

Higher bone density was significantly associated with decreased odds of mortality at 6 months (OR 0.69 per 100 HU increase in bone density; 95% CI 0.49–0.96 *p* < 0.027). However, in a multivariable analysis, the association of bone density with mortality lost its statistical significance after adjusting for other covariables including severity score (Table 2). Also, among survivors at hospital discharge, reduced bone density was significantly associated with decreased odds of disability (OR 0.52 per 100 HU; 95% CI 0.37–0.74; *p* < 0.001). Multivariable modeling demonstrated that this association was no longer significant after adjusting for different covariables including severity score (Table 2).

### Association of admission subcutaneous adipose tissue (SAT) with outcomes

There was no statistically significant association between SAT and survival at 6 months (OR 0.99; 95% CI 0.97–1.01; *p =* 0.19) or disability at hospital discharge (OR 1.01; 95% CI 0.99–1.03; *p =* 0.13).

## Discussion

In this prospective cohort study of critically ill patients, we found a significant association of larger admission erector spinae muscle (ESM) area with higher 6-month survival. Consistent with the antigravity effect of erector spinae muscles which is indispensable to maintain the upright posture needed for locomotion, greater ESM area was also associated with regaining independent function at discharge versus the need for assisted living. This association with disability at discharge was not found in our previous analysis of the non-locomotor pectoralis muscle area. The associations of muscle area with survival persisted even after adjusting for severity score, age, albumin, exercise limitation represented by modified Medical Research Council dyspnea score (mMRC), and other variables. By contrast, the association between muscle mass and disability at discharge was not statistically significant after including associations with other variables in the multivariable analysis. Our data also indicates that while higher bone density associates with better survival and less disability at hospital discharge, these correlations become insignificant after correcting for other covariables including severity score and muscle mass. Fat area was not found associated with the mortality and disability outcomes.

While the effects of critical illness on bone density and fracture risk have been described [17, 23, 24], to our knowledge, this is the first analysis that evaluates the associations of baseline bone density with survival and disability associated with critical illness, especially accounting for other relevant body mass components. Previous evidence indicates that higher preadmission weight associates with better post-ICU status [25]; however, it is unclear which body constituent accounted for the weight’s salutatory effect in that study. Indeed, it has been reported that obesity associates with greater survival in critical illness [11, 26] and that morbid obesity does not associate with worse outcomes [14]. Therefore, it is plausible that fat, and not muscle or bone, contributes to better prognosis. While a recent retrospective study involving 25 patients suggests that baseline body composition including fat and muscle is not associated with ICU length of stay [27], no evaluation of survival was reported and the small sample size of that cohort precluded analyses combining relevant covariables. Our data suggests that subcutaneous adipose tissue has no association with outcomes and that it is the contribution of muscle to body weight rather than fat or bone that is independently associated with survival.

The main strengths of this study are the large size of our cohort and the prospective study design. In addition, the simultaneous determination of major body constituents allowed for accurate analysis of the interplay of these variables’ effects on survival and disability at discharge. The selection of the erector spinae muscle, which is easily measurable [18] and needed for locomotion [28], allows for assessment of the systemic/global effects of muscle wasting on ICU outcomes combined with disability at discharge. Furthermore, ESM area is accessible via CT chest and abdomen which expands its potential use the in the ICU setting, has both type I (oxidative) and II (glycolytic) fibers [28], and thus reflects muscle wasting even in the presence of comorbidities that demonstrate selective fiber-type atrophy [29].

Our study has some limitations. First, it was performed at a single institution, and although the admission diagnoses of our cohort were diverse, it is possible that the distribution of diagnoses is not generalizable to other ICUs. Second, CT scans were performed for a clinical indication and not as part of a study protocol. Because certain diagnoses are more frequently evaluated by chest and abdomen CT scan than others, a selection bias could not be avoided, and as shown by Tables E5, E6, E7, there were significant differences in the indications for ICU admission in our cohort versus the general census during the enrollment period. However, our study population is similar to other major studies’ in the field: a recent systematic review of prospective data involving 31 studies and 3905 patients focused on ICU-associated muscle dysfunction [30] found that 39% of ICU admissions were due to respiratory failure (40% in our study), 15% were due to sepsis (19% in our study), and average patient age was 61 years old (62 in our study). Third, while previous research has investigated lumbar muscle mass in ICU patients [31], the use of the ESM area surrogate has not been validated in this population, although it has been used in this fashion in healthy and chronically ill patients [18, 28]. Likewise, while fat/SAT measurement at the thoracic 7–8 level has not been validated for ICU patients, it has shown to be highly correlated with the thoracic adipose tissue volume and with body mass index [19]. CT scan bone density has been previously used in critical illness and other settings [21, 32], and although it has comparable performance to dual X-ray absorptiometry (DXA) scan at the femoral neck level [33], it has not been validated in the ICU setting. Fourth, because patients were enrolled after they had already developed critical illness, we had to select a surrogate measure of prehospitalization mobility status. While previous research has used simple surrogates of preadmission exercise capacity such as the patients’ relatives-reported ability to ambulate up 10 stairs before hospitalization [34], we chose the mMRC score because of its simplicity, high interobserver agreement, and adequate correlation with other scoring systems [35] (Table E8–9). We realize that the mMRC is potentially confounded by cardiopulmonary limitations and other factors. However, as many of our patients were unable to cooperate with the evaluation, volitional tests such as MRC muscle strength scores [7] or other direct muscle evaluations [36] were not feasible. Fifth, the stratification of disability at discharge was based on the discharge location which does not directly address the subject’s functional capacity at that timepoint. Future research to address the association between muscle mass and activities of daily living (ADLs) at hospital discharge will add more granularity to this issue. Sixth, the used multivariable modeling was intended to define specific associations of body constituents with outcome measures, which could imply that unmeasured confounding and residual bias cannot be excluded.

## Conclusion

In summary, our data suggests that admission ESM area and bone density of ICU patients are associated with 6-month survival and with disability at hospital discharge. Larger muscle area is the only body weight constituent that persists significantly associated with survival even after multivariable adjustments; yet it does not persist associated with disability at discharge after these adjustments. Fat area is not associated with the analyzed outcomes variables. Because these outcomes are highly relevant, we postulate that further investigation may clarify whether better muscle mass can lead to improved outcomes. Our data supports the rationale of conducting mechanistic research focused on skeletal muscle integrity to interrogate its potential benefits to improve ICU prognosis [37–44].

## Supplementary information

**Additional file 1: Table E1:** Erector spinae muscle (ESM) cross sectional area and bone density associations with age.

**Additional file 2: Table E2**: Disposition of enrolled patients.

**Additional file 3: Table E3:** Survival analysis using the Cox proportional hazard determination, both univariate (above) and multivariate (below).

**Additional file 4: Table E4:** Correction of females’ muscle sizes by ideal body weight (IBW) using Devine’s formula and adjustment factor 1.67.

**Additional file 5: Table E5:** Primary indications of ICU admission in our cohort (based on 483 images used to measure subcutaneous adipose tissue as reference) versus the general MICU census (minus the patients from our cohort) during the enrollment period.

**Additional file 6: Table E6:** Primary indications of ICU admission in our cohort (based on 483 images used to measure subcutaneous adipose tissue as reference) versus the general MICU census during the enrollment period.

**Additional file 7: Table E7:** Primary indication of chest and abdomen CT scans in our cohort, based on 483 images used to measure subcutaneous adipose tissue as reference.

**Additional file 8: Table E8**: Modified Medical Research Council (mMRC) dyspnea score.

**Additional file 9: Table E9**: mMRC determination in our cohort.

**Additional file 10: Table E10:** Intra observer muscle, bone and fat variabilities.

## Data Availability

The datasets generated during and/or analyzed during the current study are not publicly available due to IRB provisions but are available from the corresponding author on reasonable request.
