# Supplementary material for: ICU admission body composition: skeletal muscle, bone, and fat effects on mortality and disability at hospital discharge—a prospective, cohort study
Source: Crit Care. 2020 Sep 21;24:566. doi: 10.1186/s13054-020-03276-9 (PMC7507825; doi:10.1186/s13054-020-03276-9)
Supplement: Supplementary file 1 — Additional file 1: Table E1: Erector spinae muscle (ESM) cross sectional area and bone density associations with age. [file 13054_2020_3276_MOESM1_ESM.docx]

| **Table E1: Association of patients' age with muscle mass and bone density** | |  |
| --- | --- | --- |
|  | **Muscle mass (females X 1.67)** | **Bone density** |
| **Slope** | -0.1331 +/- 0.0377 | -0.1879 +/- 0.173 |
| ***r2*** | 2.75% | 21% |
| ***p* value** | *p*<0.001 | *p*<0.001 |
