# Supplementary material for: ICU admission body composition: skeletal muscle, bone, and fat effects on mortality and disability at hospital discharge—a prospective, cohort study
Source: Crit Care. 2020 Sep 21;24:566. doi: 10.1186/s13054-020-03276-9 (PMC7507825; doi:10.1186/s13054-020-03276-9)
Supplement: Supplementary file 2 — Additional file 2: Table E2: Disposition of enrolled patients. [file 13054_2020_3276_MOESM2_ESM.docx]

| **Table E2: Disposition of enrolled patients** | **Total** | **Percentage** |
| --- | --- | --- |
| **Eligible patients** | 643 | 100% |
| Enrolled patients | 507 | 78% |
| Patients discharged alive | 432 | 85% (432/507) |
| Patients independent at discharge | 267 | 62% (267/432) |
| Patients not independent at discharge | 165 | 38% (165/432) |
| **Status at 6 months post-enrollment** |  |  |
| Patients alive | 317 | 62% (317/507) |
| Patients dead | 168 | 33% (168/507) |
| Patients with unknown survival status | 22 | 5% (22/507) |
