# Supplementary material for: ICU admission body composition: skeletal muscle, bone, and fat effects on mortality and disability at hospital discharge—a prospective, cohort study
Source: Crit Care. 2020 Sep 21;24:566. doi: 10.1186/s13054-020-03276-9 (PMC7507825; doi:10.1186/s13054-020-03276-9)
Supplement: Supplementary file 4 — Additional file 4: Table E4: Correction of females’ muscle sizes by ideal body weight (IBW) using Devine’s formula and adjustment factor 1.67. [file 13054_2020_3276_MOESM4_ESM.docx]

| **Table E4: raw or adjusted *p* values of ESM/outcomes** | |  |
| --- | --- | --- |
|  | **Disability at discharge** | **6-months survival** |
| **Raw ESM area** | *p*<0.001 | *p*<0.001 |
| **ESM area/IBW** | *p*=0.03 | *p*<0.001 |
| **ESM area X 1.67** | *p*=0.01 | *p*<0.001 |
