# Supplementary material for: ICU admission body composition: skeletal muscle, bone, and fat effects on mortality and disability at hospital discharge—a prospective, cohort study
Source: Crit Care. 2020 Sep 21;24:566. doi: 10.1186/s13054-020-03276-9 (PMC7507825; doi:10.1186/s13054-020-03276-9)
Supplement: Supplementary file 5 — Additional file 5: Table E5: Primary indications of ICU admission in our cohort (based on 483 images used to measure subcutaneous adipose tissue as reference) versus the general MICU census (minus the patients from our cohort) during the enrollment period. [file 13054_2020_3276_MOESM5_ESM.docx]

| **Table E5: Primary indications for ICU admission in our cohort versus patients with no CT chest performed at admission** | | | | |  |
| --- | --- | --- | --- | --- | --- |
|  | **Patients with no CT at admission (n=1615)** | | **Our cohort**  **(n=483)** | | ***p*=** |
| **Indication** | **Number** | **%** | **Number** | **%** |  |
| Non-respiratory sepsis | 530 | 32 | 92 | 19 | **<0.001** |
| Respiratory failure | 291 | 18 | 194 | 40 | **<0.001** |
| Metabolic cause including DKA | 197 | 12 | 16 | 3.5 | **<0.001** |
| Hemorrhagic shock | 125 | 8 | 40 | 8 | 0.7 |
| Stroke/seizure/altered mental status | 139 | 9 | 23 | 5 | **0.05** |
| Cardiovascular decompensation | 96 | 6 | 16 | 3.5 | **0.023** |
| Pulmonary embolism | 85 | 5 | 70 | 14.5 | **<0.001** |
| Trauma | 24 | 1.5 | 11 | 2 | 0.23 |
| Other causes | 128 | 8 | 21 | 4.5 | **0.0071** |
