# Supplementary material for: ICU admission body composition: skeletal muscle, bone, and fat effects on mortality and disability at hospital discharge—a prospective, cohort study
Source: Crit Care. 2020 Sep 21;24:566. doi: 10.1186/s13054-020-03276-9 (PMC7507825; doi:10.1186/s13054-020-03276-9)
Supplement: Supplementary file 6 — Additional file 6: Table E6: Primary indications of ICU admission in our cohort (based on 483 images used to measure subcutaneous adipose tissue as reference) versus the general MICU census during the enrollment period. [file 13054_2020_3276_MOESM6_ESM.docx]

| **Table E6: Primary indications for ICU admission in our cohort versus the entire census during the enrollment period** | | | | |  |
| --- | --- | --- | --- | --- | --- |
|  | **Entire census (n=2098)** | | **Our cohort (n=483)** | | ***p*=** |
| **Indication** | **Number** | **%** | **Number** | **%** |  |
| Non-respiratory sepsis | 622 | 30 | 92 | 19 | **<0.001** |
| Respiratory failure | 485 | 23 | 194 | 40 | **<0.001** |
| Metabolic cause including DKA | 213 | 10 | 16 | 3.5 | **<0.001** |
| Hemorrhagic shock | 165 | 8 | 40 | 8 | **<0.001** |
| Stroke/seizure/altered mental status | 162 | 7 | 23 | 5 | **<0.001** |
| Cardiovascular decompensation | 112 | 5 | 16 | 3.5 | **<0.001** |
| Pulmonary embolism | 155 | 7 | 70 | 14.5 | **<0.001** |
| Trauma | 35 | 1.7 | 11 | 2 | **<0.001** |
| Other causes | 149 | 7 | 21 | 4.5 | **0.0071** |
