# Supplementary material for: ICU admission body composition: skeletal muscle, bone, and fat effects on mortality and disability at hospital discharge—a prospective, cohort study
Source: Crit Care. 2020 Sep 21;24:566. doi: 10.1186/s13054-020-03276-9 (PMC7507825; doi:10.1186/s13054-020-03276-9)
Supplement: Supplementary file 7 — Additional file 7: Table E7: Primary indication of chest and abdomen CT scans in our cohort, based on 483 images used to measure subcutaneous adipose tissue as reference. [file 13054_2020_3276_MOESM7_ESM.docx]

| **Table E7: Primary indications for CT chest or abdomen (total 483)** | | | | |  |
| --- | --- | --- | --- | --- | --- |
| **Indication** | **Number** | | **%** | | |
| Suspected pulmonary embolism | | 130 | | 27 | |
| Respiratory distress | | 77 | | 16 | |
| Suspected pneumonia or atelectasis | | 71 | | 14 | |
| Trauma of chest or abdomen | | 49 | | 10 | |
| Suspected abdominal or pelvic infection | | 39 | | 8 | |
| Suspected cavitary lesion or effusion | | 36 | | 7 | |
| Suspected metastasis or mediastinal mass | | 31 | | 7 | |
| Suspect vascular abnormality | | 17 | | 3.5 | |
| Hemoptysis | | 16 | | 3.5 | |
| Unspecified chest X ray abnormality | | 12 | | 2 | |
| Suspected pneumomediastinum /pneumothorax | | 5 | | 1 | |
