# Supplementary material for: ICU admission body composition: skeletal muscle, bone, and fat effects on mortality and disability at hospital discharge—a prospective, cohort study
Source: Crit Care. 2020 Sep 21;24:566. doi: 10.1186/s13054-020-03276-9 (PMC7507825; doi:10.1186/s13054-020-03276-9)
Supplement: Supplementary file 8 — Additional file 8: Table E8: Modified Medical Research Council (mMRC) dyspnea score. [file 13054_2020_3276_MOESM8_ESM.docx]

| **Table E8: mMRC dyspnea score** |  |
| --- | --- |
| **1:** Dyspnea when strenuous exercise |  |
| **2:** Dyspnea when hurrying on the level or walking up a slight hill |  |
| **3:** Walks slower than most people on the level, or stops after a mile or so, or stops after 15 min walking at own pace | |
| **4:** Stops for breath after walking 100 yards (91m), or after few minutes on level ground |  |
| **5:** Too dyspneic to leave the house or breathless when dressing |  |
