# Supplementary material for: ICU admission body composition: skeletal muscle, bone, and fat effects on mortality and disability at hospital discharge—a prospective, cohort study
Source: Crit Care. 2020 Sep 21;24:566. doi: 10.1186/s13054-020-03276-9 (PMC7507825; doi:10.1186/s13054-020-03276-9)
Supplement: Supplementary file 9 — Additional file 9: Table E9: mMRC determination in our cohort. [file 13054_2020_3276_MOESM9_ESM.docx]

| **Table E9: mMRC determination** | **Total** | **Percentage** |
| --- | --- | --- |
| Patients responding to mMRC at the time of consent: | 288 | 57% |
| Patient's surrogate responding to mMRC at the time of consent: | 219 | 43% |
| **Out of the surrogates' responding group:** |  |  |
| Patients who never regained capacity to provide mMRC score: | 128 | 59% (128/219) |
| Patients who regained capacity to provide mMRC score | 91 | 41% (91/219) |
| Patients who provided mMRC score similar from initially obtained: | 82 | 90% (82/91) |
| Patients who provided mMRC score different from initially obtained: | 9 | 10% (9/91) |
