# Supplementary material for: ICU admission body composition: skeletal muscle, bone, and fat effects on mortality and disability at hospital discharge—a prospective, cohort study
Source: Crit Care. 2020 Sep 21;24:566. doi: 10.1186/s13054-020-03276-9 (PMC7507825; doi:10.1186/s13054-020-03276-9)
Supplement: Supplementary file 10 — Additional file 10: Table E10: Intra observer muscle, bone and fat variabilities. [file 13054_2020_3276_MOESM10_ESM.docx]

| **Table E10: Intra observer variability of fat, muscle and bone measurements performed at least 6 months apart** | | | | | | | | | | | |  |  | | |
| --- | --- | --- | --- | --- | --- | --- | --- | --- | --- | --- | --- | --- | --- | --- | --- |
| **Patient number** | **SAT 1** | **SAT 2** | | | **ESM right** | **ESM left** | **ESM aggregate 1** | **ESM right** | | **ESM left** | **ESM aggregate 2** | **Bone 1** | **Bone 2** | | |
| 7 | 21868 | 22823 | | | 1778 | 1511 | 32.89 | 1730 | | 1584 | 33.14 | 257.8 | 250.3 | | |
| 22 | 29014 | 30126 | | | 1038 | 1087 | 21.25 | 931 | | 1194 | 21.25 | 84.6 | 77.4 | | |
| 56 | 16602 | 16420 | | | 1257 | 1139 | 23.96 | 1243 | | 1171 | 24.14 | 200.5 | 191.7 | | |
| 64 | 49222 | 50097 | | | 1183 | 1148 | 23.31 | 1068 | | 1183 | 22.51 | 173.7 | 172.9 | | |
| 73 | 29386 | 29386 | | | 1232 | 1171 | 24.03 | 1258 | | 1266 | 25.24 | 122.3 | 119 | | |
| 84 | 39486 | 41640 | | | 1428 | 1412 | 28.4 | 1387 | | 1494 | 28.81 | 158.3 | 176.2 | | |
| 92 | 22948 | 24020 | | | 1753 | 1769 | 35.22 | 1710 | | 1720 | 34.3 | 117.3 | 131.1 | | |
| 107 | 10312 | 9660 | | | 1654 | 1537 | 31.91 | 1889 | | 1619 | 35.08 | 131.9 | 131.8 | | |
| 121 | 5767 | 6161 | | | 1333 | 1377 | 27.1 | 1541 | | 1612 | 31.53 | 121.4 | 129.8 | | |
| 131 | 16267 | 16747 | | | 1555 | 1610 | 31.65 | 1926 | | 2001 | 39.27 | 158.3 | 154.4 | | |
| 143 | 32024 | 32373 | | | 1355 | 1215 | 25.7 | 1201 | | 1209 | 24.1 | 64.3 | 72.5 | | |
| 157 | 18857 | 19424 | | | 1888 | 1659 | 35.47 | 1979 | | 1683 | 36.62 | 140.5 | 142.9 | | |
| 166 | 10455 | 9759 | | | 1218 | 1130 | 23.48 | 1179 | | 1344 | 25.23 | 203.7 | 195.9 | | |
| 177 | 8742 | 8769 | | | 676 | 692 | 13.68 | 702 | | 755 | 14.57 | 112 | 111.9 | | |
| 188 | 15073 | 14916 | | | 1380 | 1424 | 28.04 | 1424 | | 1465 | 28.89 | 69.2 | 69.5 | | |
| 198 | 20565 | 20927 | | | 1123 | 1036 | 21.59 | 1072 | | 1105 | 21.77 | 212.3 | 221.1 | | |
| 229 | 25335 | 24571 | | | 1865 | 1781 | 36.46 | 1932 | | 1814 | 37.46 | 117.7 | 112.3 | | |
| 234 | 6001 | 5467 | | | 988 | 975 | 19.63 | 1026 | | 1123 | 21.49 | 122.2 | 121.6 | | |
| 251 | 13499 | 13876 | | | 1063 | 1088 | 21.51 | 1050 | | 1026 | 20.76 | 232.3 | 230.6 | | |
| 268 | 27127 | 26447 | | | 1146 | 1039 | 21.85 | 1180 | | 1024 | 22.04 | 42.7 | 45.4 | | |
|  |  |  | | |  |  |  |  | |  |  |  |  | | |
|  | **Diff fat**  **0.253 cm2 (+/- 3.6)** | | |  |  | **Diff muscle**  **1.054 cm2 +/-2.042** | |  |  |  |  | **Diff bone**  **0.76 HU +/-18.2** | | |  |
|  | **p=0.9** | |  | |  | **p=0.6** |  |  | |  |  | **p=0.96** | |  | |
