# Supplementary material for: ICU admission body composition: skeletal muscle, bone, and fat effects on mortality and disability at hospital discharge—a prospective, cohort study
Source: Crit Care. 2020 Sep 21;24:566. doi: 10.1186/s13054-020-03276-9 (PMC7507825; doi:10.1186/s13054-020-03276-9)
Supplement: Supplementary file 10 [file 13054_2020_3276_MOESM3_ESM.docx]

**Table E3: Survival analysis**						
**Univariate Cox Proportional Hazard**						
		**Term**	**Risk Ratio**	**Lower 95%**	**Upper 95%**	***p***
		Sex M/F	0.8	0.14	0.58	0.14
		Age (per decade)	1.23	1.11	1.36	**<0.001**
		SOFA	1.16	1.12	1.2	**<0.001**
		APACHE II	1.07	1.05	1.08	**<0.001**
		Bone density (per 100 HU)	0.76	0.58	0.98	**0.04**
		ESMCSA (muscle area, per cm2)	0.96	0.95	0.98	**<0.001**
		Fat (per 10 cm2)	0.99	0.98	1	0.15
		mMRC	2.67	1.8	3.97	**<0.001**
		Albumin	0.44	0.36	0.55	**<0.001**
		not independent on admission	1.39	1.02	1.91	**0.04**
		Cancer	2.77	2.02	3.77	**<0.001**
		Chronic Pulmonary Dis.	1.11	0.81	1.51	0.52
		CHF	1.31	0.9	1.86	0.15
		End Stage Renal Disease	1.69	1.03	2.63	**0.04**
		Chronic Steroids	1.11	0.77	1.56	0.58
**Multivariable Cox Proportional Hazard with backward stepping for variable selection with p<0.1 criteria.**						
		**Term**	**Risk Ratio**	**Lower 95%**	**Upper 95%**	***p***
		Age (per decade)	1.13	1	1.29	**0.046**
		SOFA	1.15	1.1	1.2	**<0.001**
		ESMCSA (muscle area, per cm2)	0.98	0.97	0.9995	**0.044**
		mMRC	1.16	1.04	1.3	**0.011**
		Albumin	0.63	0.48	0.82	**<0.001**
		cancer	2.42	1.7	3.42	**<0.001**
